# Primary Anterior Mediastinal Cholesterol Granuloma: A Rare Case in a Young Woman and Literature Review

**DOI:** 10.2174/0115734056413783251020183226

**Published:** 2025-10-29

**Authors:** Xuan Qiu, Jialan Huang, Hua Ye, Shuying Luo, Qin Zhang, Hong Yu

**Affiliations:** 1 Department of Radiology, Affiliated Hospital of Zunyi Medical University, Medical Imaging Center of Guizhou Province, No. 149 Dalian Road, Zunyi city, Guizhou province, 563003, China; 2 Department of Pathology, Affiliated Hospital of Zunyi Medical University, No. 149 Dalian Road, Zunyi city, Guizhou province, 563003, China

**Keywords:** Mediastinal cholesterol granuloma, Mediastinal mass, MRI, PET, Multimodal imaging, Chronic granulomatous inflammation

## Abstract

**Background:**

Mediastinal cholesterol granuloma (MCG) is an exceedingly rare condition, with a limited number of cases reported worldwide. The clinical and imaging characteristics of MCG remain poorly understood and often lead to misdiagnosis. This case report of a young female patient contributes to the literature by summarizing the clinical features, imaging findings, and differential diagnosis of MCG in a demographic category rarely described in previous reports.

**Case Description:**

A 30-year-old female with a history of community-acquired pneumonia, pulmonary tuberculosis (cured), and syphilis was incidentally found to have an anterior mediastinal mass on imaging. This patient had no history of trauma or other risk factors related to the onset of MCG. Meanwhile, the gender and age characteristics were also different from those commonly seen in the literature. Surgical resection at our hospital confirmed the diagnosis of thymic cholesterol granuloma. Literature review identified 24 reported cases of MCG, predominantly in older males (94.74%; average age, 58.3 years), with a geographic distribution across Europe, East Asia, and North America (36.8%, 31.6%, and 26.3%, respectively). Notably, three of the cases involved young and middle-aged patients with a history of chest trauma. The imaging features varied, with magnetic resonance imaging (MRI) showing low signal (indicating cholesterol crystals) or high signal intensity (due to methemoglobin) on T1/T2-weighted images. Positron emission tomography (PET) scans typically revealed high uptake signals attributed to chronic granulomatous inflammation.

**Conclusion:**

MCG is a rare anterior mediastinal lesion with nonspecific imaging features. A history of dyslipidemia or chest trauma combined with compatible imaging findings should prompt consideration of MCG in the differential diagnosis. The possibility of MCG should also be considered in young women with a history of tuberculosis or syphilis. This case highlights the importance of recognizing atypical presentations of MCG to reduce misdiagnoses and guide appropriate management.

## 1 INTRODUCTION

Mediastinal cholesterol granuloma (MCG) is a rare benign disease caused by the deposition of cholesterol crystals in the mediastinum, especially the anterior mediastinum [1]. The cause of its formation is still unclear, but it may be related to dyslipidemia or chronic inflammation [2]. Most patients exhibit no specific symptoms, and the condition is often detected incidentally during imaging tests or surgeries. Diagnosis is primarily based on imaging modalities, while surgery remains the mainstay of curative treatment. To date, only a few cases have been reported in the literature. Here, we report a case of MCG in a 30-year-old female who was suspected of having a malignant lesion based on clinical and imaging evaluation. In addition, we review previously reported cases of MCG.

## 2 CASE PRESENTATION

A 30-year-old female presented to our hospital for evaluation of an anterior mediastinal mass. Her history indicated a prior diagnosis of community-acquired pneumonia at a local hospital, where she received treatment for symptoms, including cough and expectoration. During this treatment, a chest computed tomography (CT) scan revealed the anterior mediastinal mass. Apart from her pneumonia symptoms, she reported no specific complaints related to the mass, such as pressure effects or mass-associated symptoms. The patient tested positive for *T. pallidum* antibodies and had been diagnosed with tuberculosis 10 years ago, which was cured with standard medications. She denied exposure to toxic, radioactive, or irritating substances, and no environmental or dietary risk factors for infectious diseases (*e.g*., tuberculosis or syphilis) were identified. The patient's physical and laboratory examination results were normal, with no ptosis or other relevant findings. As her cough and expectoration resolved after anti-infective treatment at the outside hospital, and the chest CT showed no residual pneumonia, the thoracic surgery department omitted pulmonary function tests (PFTs) from the preoperative assessment. A lipid profile was not performed; however, serum biochemistry test and complete blood count were normal. It should be noted that large mediastinal tumors can cause restrictive ventilatory dysfunction due to mass effect, potentially altering pulmonary function tests. Dyslipidemia may also contribute to MCG development. Unfortunately, these specific assessments (pulmonary function and lipid profile) were not obtained.

**Fig. (1):**
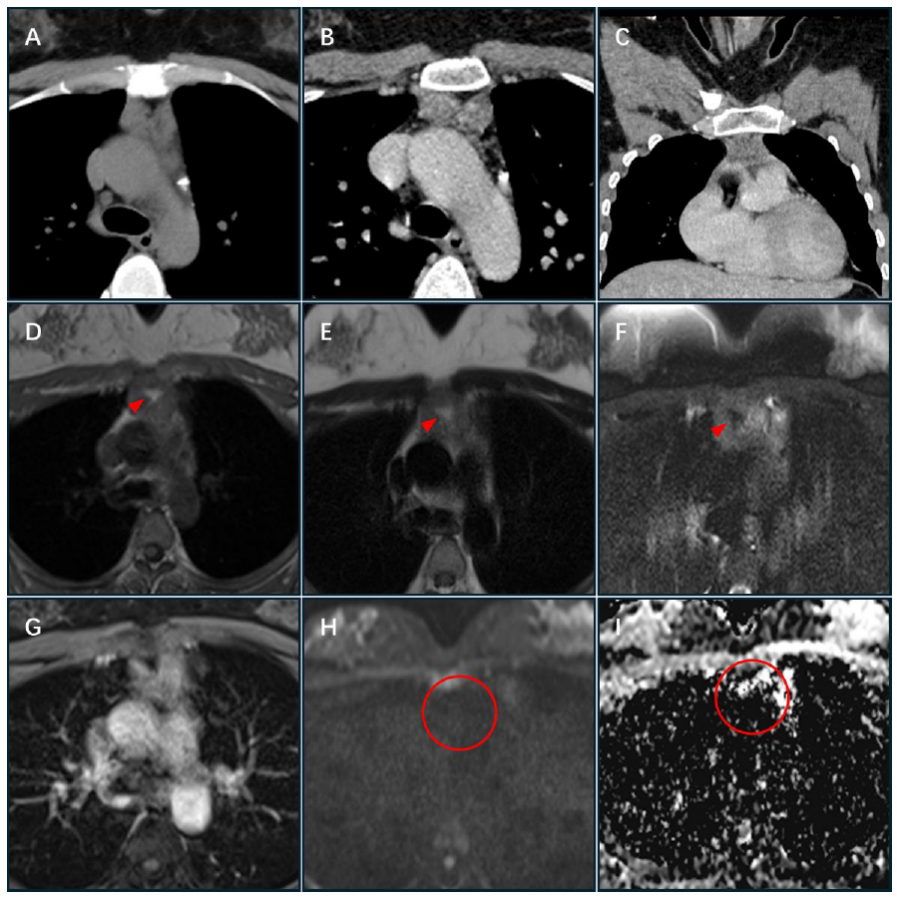
(**A-C**) Enhanced chest CT showed a nodular mass with mild enhancement located in the anterior superior mediastinum. (**D, E**) T1- and T2-weighted MRI showed a mass with low signal in the edge and a high signal in the center (arrow). (**F**) After fat-suppression sequence imaging, the central high signal area of the mass became low signal (arrow). (**G**) The enhanced scan showed significant enhancement. (**H, I**) The DWI sequence was a low signal, and the ADC sequence was a partially high signal (circle). CT: Computed tomography; MRI: Magnetic resonance imaging; DWI: Diffusion weighted imaging; ADC: Apparent diffusion coefficient. **Note:** CT: Computed tomography; MRI: Magnetic resonance imaging; DWI: Diffusion weighted imaging; ADC: Apparent diffusion coefficient.

CT revealed an irregular mass in the anterior mediastinum, approximately 32 mm × 18 mm in size, with clear boundaries, uneven soft-tissue density, and internal fat density. The average CT value of the plain scan was approximately 20 Hounsfield unit (HU), and the enhanced scan showed obvious enhancement leading to the CT diagnosis of thymoma (Fig. **1A**-**C**). Magnetic resonance imaging showed a rounded mass in the anterior mediastinum with overall low signal intensity, but an irregular high-signal area in the center of the mass on T1- and T2-weighted images (Fig. **1D**, **E**). Fat-suppression sequence imaging was performed to distinguish the internal components of the mass. The results showed that the edge of the mass did not change; however, the central high-signal area became a low-signal area (Fig. **1F**), indicating that it was a lipid component. Consistent with CT findings, the mass showed obvious enhancement on the enhanced scan (Fig. **1G**). Additionally, the mass showed low signal on diffusion-weighted imaging (DWI) and partially high signal on apparent diffusion coefficient (ADC) sequence, indicating that some water molecules within the tumor had unrestricted dispersion (Fig. **1H**, **I**). Based on the above multimodal imaging findings, the malignancy was suspected.

**Fig. (2):**
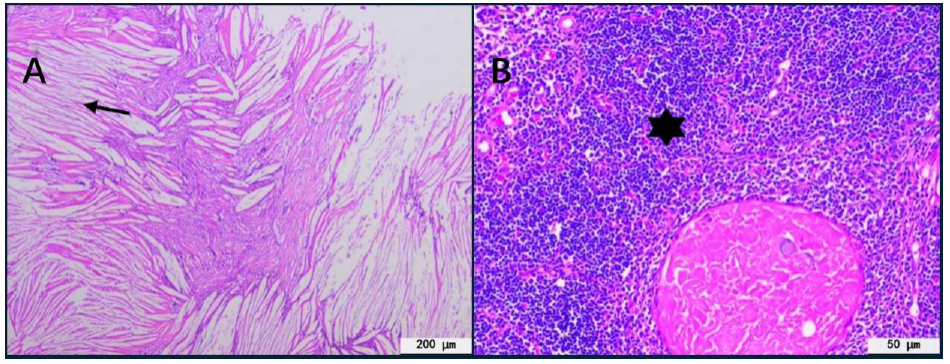
(**A**) The hematoxylin-eosin stained section displays a multitude of distinctive spindle-shaped cholesterol crystals. These crystals are enveloped by a rim of short spindle-shaped cells, as indicated by arrows. The surrounding area is populated by macrophages, which are interspersed with minor calcifications. (**B**) This section reveals a substantial amount of granulation tissue, marked by an asterisk. Within this tissue, a few nuclear foreign body giant cells are discernible, contributing to the formation of the granulomatous structure.

Based on the above multimodal imaging findings, the anterior mediastinal mass was suspected to be malignant. To establish a definitive diagnosis and provide therapeutic intervention, the patient underwent complete subxiphoid thoracoscopic resection of the mediastinal tumor. The resected specimen was submitted for histopathological examination. The mass was located in the anterior mediastinum, measuring 3 cm × 1.5 cm × 1.5 cm; the gross specimen had broken greyish-white-yellow tissue, with a greyish-white nodule visible within. On sectioning, the interior was cystic and solid with a yellow jelly-like substance. Microscopic pathological examination revealed short spindle-shaped cells arranged in bundles or crosses, with well-defined nuclei and abundant eosinophilic cytoplasm, accompanied by calcification and cleft-like cholesterol crystal formation. Hemorrhagic necrotic tissue was observed in some areas surrounded by multinucleated foreign body giant cells and a large amount of granulation tissue. Therefore, pathological examination confirmed thymic cholesterol granuloma (Fig. **2**). The patient was discharged from the hospital after the successful operation and was followed up for 4 years without recurrence.

## 3 LITERATURE REVIEW

For conducting the literature review, the PubMed database was extensively searched for relevant articles, and the language of the articles was limited to English. Reports from January 2000 to September 2024 were considered, and the keywords “cholesterol granuloma” or “cholesteroloma” were used. The keywords “OR” and “AND” were used individually. A total of 16 articles reporting 24 cases were included in the literature review. A flowchart of the literature screening process is shown in Fig. (**3**). For each case, data on the first author, year of publication, patient age/sex/background, site, size, radiological findings, and country of origin were collected (Table **1**).

**Fig. (4):**
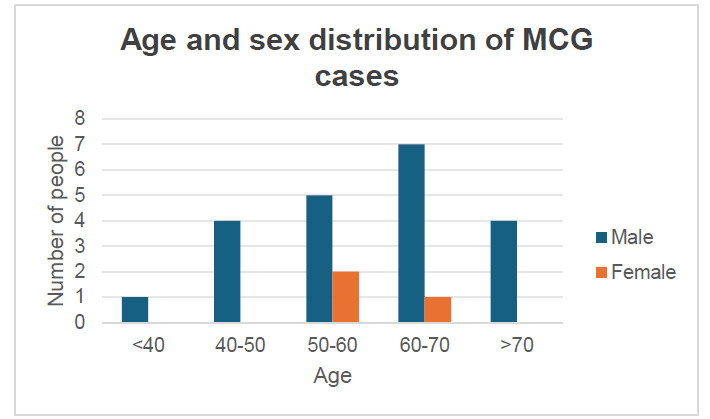
The age and gender distribution of cholesterol granuloma. Most cases are reported in middle-aged and elderly people, with males accounting for the majority.

We reviewed 24 previously reported cases of MCG, all of which involved the anterior mediastinum, with a male-to-female ratio of 21:3, an age of onset ranging from 25-75 years (mean age, 58.3 years), and a history of trauma in three cases (Fig. **4**). MRI revealed mainly low signal on T1- and T2-weighted images, related to the presence of cholesterol crystals within the lesion. High signal intensity on T1- and T2-weighted images has also been reported, related to the presence of methemoglobin in the mass. On PET imaging, the lesions showed high uptake signals because of granulomatous inflammation, characterized by the aggregation of macrophages and lymphocytes and increased ^18^F-FDG uptake. On pathological examination, almost all lesions were located in the thymus or had thymic tissue visible in their periphery; therefore, Weissferdt suggested that the lesion could also be named “cholesteroloma” [3].

## 4 DISCUSSION

Cholesterol granuloma is a granulomatous lesion caused by the reaction of the body to cholesterol crystals. The pathological manifestation is the presence of a large number of cholesterol crystals, accompanied by infiltration of macrophages and foreign body giant cells and formation of fibrous granulation tissue. Calcification and ossification may occur occasionally [4]. Cholesterol granulomas can occur in any area of the body in response to the deposition of cholesterol crystals. They are predominantly found in the temporal bone and have been reported in the kidneys, mammary glands, peritoneum, parotid glands, testes, lungs, liver, and spleen [5]. Cholesterol granulomas occurring in the anterior mediastinum are relatively rare. The main pathogenesis of this disease is trauma and inflammation. Chronic trauma or inflammation releases cholesterol from the cell membranes of denatured red blood cells to form crystals, thus stimulating the foreign body giant cell reaction and leading to the formation of cholesterol granulomas [6]. Notably, three of the 24 patients reported in the reviewed literature had a history of trauma or frequent blunt force injuries to the chest (such as in football players), suggesting that blunt trauma to the chest may cause the release of cholesterol crystals. Deposition of cholesterol crystals can have a multifactorial etiology, including the presence of dyslipidemia and underlying conditions, such as hypertension and vascular disease, which increase the likelihood of cholesterol crystal deposition [2]. MCGs may also result from the rupture of a benign cystic thymic tumor [3, 7].

The patient in our case had a history of tuberculosis and syphilis, raising the question of whether this could have affected the metabolic processes in the body and led to the development of MCG. Alternatively, the medications used to treat syphilis and tuberculosis could also have contributed to the disease development. This aspect needs to be explored further.

Clinical manifestations of MCGs are related to their location and size, and are often dominated by occupational effects. Most MCGs are asymptomatic; however, a few patients may have symptoms, such as coughing or dyspnea, which are mostly incidental findings. The diagnosis relies on imaging and histopathological examinations. As this disease is rare and often recognized as malignant on imaging, surgical excision is the preferred management. However, if the disease is benign, then surgical excision is not necessary.

In this case, the patient was a young female, which contrasts with the common demographic pattern reported in the literature, where the onset has been reported to occur in middle-aged or elderly male patients. Additionally, the patient had a history of tuberculosis and syphilis. Whether the pathophysiological processes of these diseases involve factors contributing to MCG development requires further investigation.

Considering these factors, MCG should be included in the differential diagnosis of anterior mediastinal masses. In middle-aged or elderly male patients, particularly those with dyslipidemia or a history of chronic chest trauma, accurate correlation between imaging findings and clinical data may facilitate the diagnosis of MCG. This approach can help avoid unnecessary interventions.

### 4.1 Differential Diagnosis

Current English-language reports on MCG indicate that pathologically confirmed lesions are confined to the thymus, with no verified cases identified in other mediastinal regions. The pathogenesis of MCG remains poorly understood, but potential contributing factors may include dyslipidemia, chronic inflammation, or increased vulnerability of the anterior mediastinum to trauma [2].

Common occupying lesions of the anterior mediastinum include thymoma, lymphoma, and intrathoracic lymph node tuberculosis.

Thymoma is the most common primary tumor of the anterior mediastinum; therefore, it should be considered first when diagnosing an anterior mediastinal mass. Thymoma on MRI shows low signal intensity on T1-weighted images and high signal on T2-weighted images; it often appears mildly enhanced in the enhancement scans. It is commonly associated with myasthenia gravis. When thymoma turns malignant, CT and MRI show necrosis, hemorrhage, and cystic changes inside the mass. Additional malignant features, such as unclear border and invasion of the surrounding structures, as well as high uptake on PET, are also observed.

Lymphoma shows mild enhancement on MRI enhanced scan, and obvious diffusion limitation with a low ADC value on DWI. Lymphomas, when growing, tend to encircle blood vessels without invading them; this is called the “vascular floating sign” and can be an important characteristic feature of lymphoma.

Intrathoracic lymph node tuberculosis is rare, with imaging features similar to those of MCG; therefore, it is included in the differential diagnosis of MCG. Intrathoracic lymph node tuberculosis may present as single or multiple intramediastinal isointense masses with common internal calcifications and mild enhancement on an enhancement scan. It is sometimes associated with intrapulmonary tuberculosis or tuberculosis of other body systems.

## CONCLUSION

In conclusion, rare diseases, such as MCG, should be included in the differential diagnosis of patients with an anterior mediastinal mass. Diagnosing thymic MCG based on imaging alone is difficult, and the likelihood of its occurrence increases if associated with hyperlipidemia or a history of chest trauma.

## Figures and Tables

**Fig. (3):**
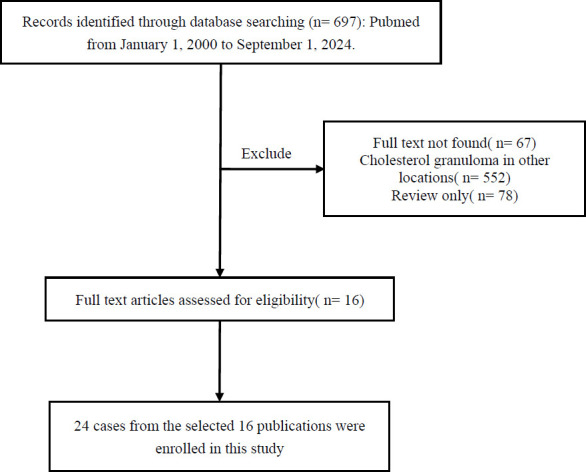
The flow chart of the literature screening process for mediastinal cholesterol granuloma.

**Table 1:** Previously reported cases of mediastinal cholesterol granuloma.

Author (year)/Refs.	Age/ Sex	Background		Site	Size (cm)/Mono or Multifocal	Radiological Findings	Country
Matsuda R, *et al*. [8] (2024)	49- 61/ M,F	Fever		Anterior	1.7×1.2; 2.3×1.6; 5.1×2.1; 3.8×4.0; 1.7×1.2/mono and multifocal	CT: slight contrast enhancement and without enhancement; PET: high uptake and low uptake	Japan
Ludoski M, *et al*. [9] (2024)	46/ M	Dyslipidemia		Anterior	11×4/mono	No data	Serbia
Suzuki M, *et al*. [4] (2023)	45/ M	Dyslipidemia and fatty liver disease		Anterior	4.2/multifocal	CT: slight contrast enhancement and spotty calcification; MRI: low signal intensity on both T1- and T2-weighted images and DWI; PET: high uptake	Japan
Hongo T, *et al*. [1] (2023)	43/ M	Long history of playing rugby		Anterior	2/mono	CT: slight contrast enhancement; MRI: low signal intensity on both T1- and T2-weighted images; PET: an increased uptake	Japan
Hendrikx B, *et al*. [10] (2022)	68/ M	Smoking and alcohol abuse		Anterior	2.5×2.0/multifocal	CT: well-delineated, lobulated soft tissue mass; PET: moderate uptake	Belgium
Manabe T, *et al*. [2] (2020)	70/ M	Dyslipidemia, hypertension		Anterior	4.2×2.6; 3.2×3.0; 2.6×2.6; 2.3×2.3/multifocal	CT: slight contrast enhancement, spotty calcification	Japan
Takeda T, *et al*. [11] (2020)	65/ M	None		Anterior	3/mono	CT: cystic lesion with a thickened wall and a small solid nodular lesion adjacent to the cyst; PET: high uptake	Japan
Paep KD, *et al*. [12] (2020)	57/ M	Smoking and using alcohol on a routine basis		Anterior	4.2×2.5/mono	PET: high uptake	Belgium
Nagata S, *et al*. [7] (2018)	56/ F	Dyslipidemia		Anterior	3.5/multifocal	CT: well-circumscribed cyst and an adjacent 10-mm nodule; PET: increased uptake only in the 10-mm nodule	Japan
Drury NE, *et al*. [13] (2017)	74/ M	Smoking and history of paroxysmal atrial fibrillation and COPD		Anterior	3.2/mono	CT: coarse calcification; PET: significant uptake	UK
Ghigna MR, *et al*. [14] (2016)	53/ M	None		Anterior	5×3/mono	No data	France
	25/ M	Car racer		Anterior	4×2.5/mono	No data	France
Weissferdt A, *et al*. [3] (2015)	58-71/ M	Cough and dyspnea		Anterior	2-6/mono	No data	USA
Krishnan TR, *et al*. [15] (2013)	65/ M	Hypertension, medically managed pericarditis		Anterior	2/mono	No data	New Zealand
Ezzat TF, *et al*. [16] (2012)	75/ M	Dyslipidemia, remote motor vehicle collision		Anterior	3/mono	No data	Canada
Fujimoto K, *et al*. [6] (2007)	62/ M	Myocardial infarction, thrombosis in the left atrium, and hyperlipidemia		Anterior	2×1.9/mono	CT: slight contrast enhancement and spotty calcification; MRI: low signal intensity on both T1- and T2-weighted images; PET: significant uptake	Japan
Luckraz H, *et al*. [17] (2006)	74/ M	None		Anterior	2.7×2.0/mono	No data	UK

## Data Availability

The data and supportive information are available within the article.

## LIST OF ABBREVIATIONS

MCG: Mediastinal cholesterol granuloma
CT: Computed tomography
PFTs: Pulmonary function tests
HU: Hounsfield unit
DWI: Diffusion-weighted imaging
ADC: Apparent diffusion coefficient
MRI: Magnetic resonance imaging
PET: Positron emission tomography
^18^F-FDG: ^18^F-fluorodeoxyglucose

## STANDARDS OF REPORTING

CARE guidelines were followed.
